# Supplementary figures and images for: “Don, doff, discard” to “don, doff, decontaminate”—FFR and mask integrity and inactivation of a SARS-CoV-2 surrogate and a norovirus following multiple vaporised hydrogen peroxide-, ultraviolet germicidal irradiation-, and dry heat decontaminations
Source: PLoS One. 2021 May 19;16(5):e0251872. doi: 10.1371/journal.pone.0251872 (PMC8133425; doi:10.1371/journal.pone.0251872)

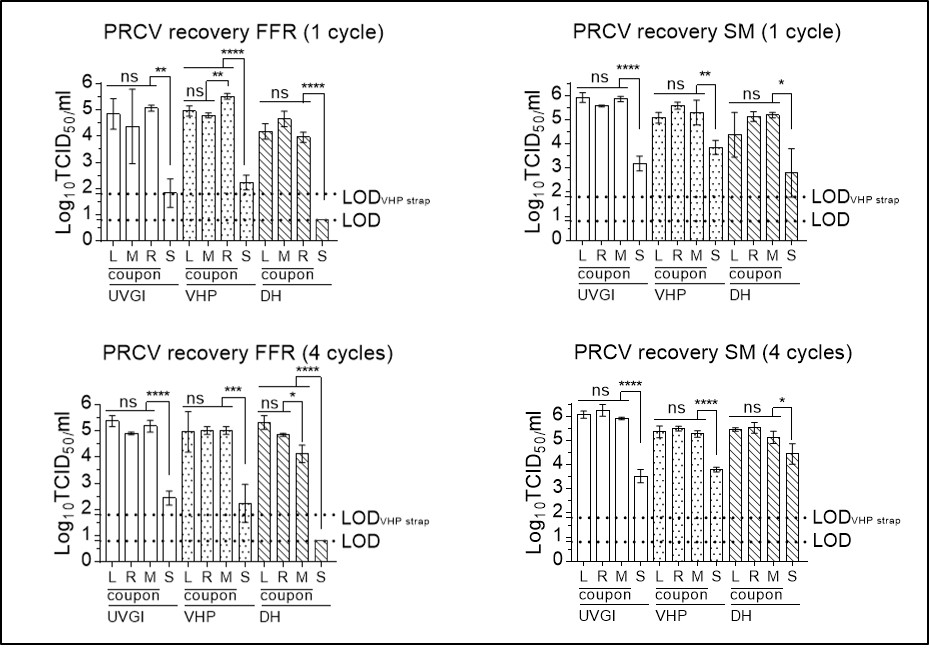

Supplement: S1 Fig — Infectious PRCV recovery was analysed in swine testicular cells. The cell culture limit of detection (LOD) was 0.80 log10 TCID50/mL (6.31×100 TCID50/mL) for all analyses except those concerning VHP-treated SM or FFR straps (1.80 log10 TCID50/mL (6.31×101 TCID50/mL)). Similar levels of virus recovery were detected for left, right and middle (L, R, M) (n = 3) coupons of FFRs and SMs; recovery efficacy of infectious virus from straps (S) (n = 3) deviated significantly in all analyses from the mean of all coupons and remained below the LOD for assays performed on DH-treated FFR straps. Mean log10 TCID50/mL and standard errors of the means are represented. P-values were computed by using a two-sided independent sample t-test to calculate differences between individual coupon values and differences between mean values of all coupons and straps, where ****P<0.0001, ***P<0.001, **P<0.01, *P<0.05, and ns. (TIF) [file pone.0251872.s001.tif]

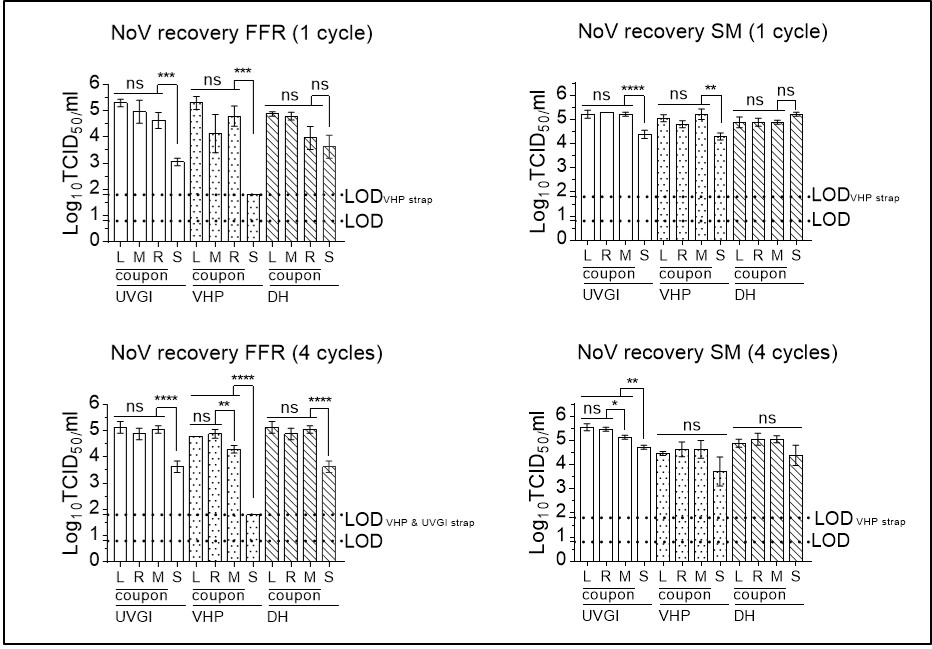

Supplement: S2 Fig — Infectious MuNoV recovery was analysed in RAW264.7 cells. The cell culture limit of detection (LOD) was 0.80 log10 TCID50/mL (6.31×100 TCID50/mL) for all analyses except those concerning VHP-treated SM- or FFR straps and UVGI-treated FFR straps (1.80 log10 TCID50/mL ((6.31×101 TCID50/mL)). Similar levels of virus recovery were detected for left, right and middle (L, R, M) (n = 3) coupons of FFRs and SMs; recovery efficacy of infectious virus from straps (S) (n = 3) deviated significantly in all analyses from the mean of all coupons (except from DH-treated straps). Mean log10 TCID50/mL and standard errors of the means are represented. P-values were computed by using a two-sided independent sample t-test to calculate differences between individual coupon values and differences between mean values of all coupons and straps, where ****P<0.0001, ***P<0.001, **P<0.01, *P<0.05, and ns. (TIF) [file pone.0251872.s002.tif]
